# 1400. Pretomanid in the Treatment of Patients with Tuberculosis in the United States: the Bedaquiline, Pretomanid and Linezolid (BPaL) Accelerated Monitoring (BAM) Project

**DOI:** 10.1093/ofid/ofab466.1592

**Published:** 2021-12-04

**Authors:** Neela Goswami, Lakshmi Praveena Peddareddy, John Jereb, Angel Colon Semidey, Elaine Darnall, Patricia Macias, Supriya Jasuja, Karen Landers, Shom Dasgupta, Margaret Oxtoby, Christopher Spitters, Lana Dov, Masa Narita, Malini DaSilva, Tracina Cropper, Cricket Gullickson, David Ashkin, Connie Haley

**Affiliations:** 1 U.S. Centers for Disease Control and Prevention, Atlanta, Georgia; 2 Puerto Rico Department of Health, San Juan, Puerto Rico; 3 Illinois Department of Public Health, Chicago, Illinois; 4 Cook County Department of Public Health, Chicago, Illinois; 5 Alabama Department of Public Health, Montgomery, Alabama; 6 Los Angeles County Department of Public Health, Los Angeles, California; 7 New York State Department of Health, Albany, New York; 8 Snohomish Health District TB program, Everett, Washington; 9 Washington State Department of Health, Olympia, Washington; 10 Seattle King County TB Program, seattle, Washington; 11 HealthPartners Institute, Minneapolis, MN; 12 Florida Department of Health, Miami, Florida; 13 Southeastern National TB Center, Gainesville, Florida

## Abstract

**Background:**

In August 2019 the U.S. FDA approved pretomanid as part of a 6-month all-oral BPaL (bedaquiline, pretomanid, and linezolid) regimen for treating pulmonary extensively drug-resistant (XDR) or treatment-intolerant or nonresponsive multidrug-resistant (MDR) tuberculosis (TB). In the study supporting approval, 89% of patients had a favorable outcome, and all reported ≥ 1 adverse event. We describe the reported use of BPaL in the United States.

**Methods:**

Using the 2020 CDC Report of a Verified Case of Tuberculosis (RVCT) MDR TB supplemental form, TB programs and providers submitted data for patients who began taking BPaL between Aug 1, 2019 and May 1, 2020, for retrospective descriptive analysis.

**Results:**

Programs and providers reported 17 TB patients aged a mean of 41 years (range 23–76) who received BPaL: 11 (65%) were male; 15 (88%) were non-U.S. born; 15 (88%) had pulmonary TB disease only; two (12%) had both pulmonary and extrapulmonary disease. Of all patients, 16 had *Mycobacterium tuberculosis* isolated from sputum and 7 (44%) had cavitary disease. The preliminary drug susceptibilities were 8 MDR patterns, 8 pre-XDR, and 1 unreported. Three patients received BPaL as their only treatment; six first received treatment for drug-susceptible TB, and eight received other regimens for MDR TB before BPaL. Eleven (65%) patients had ≥ 1 side effect reported during any TB treatment, including peripheral neuropathy (n=5), depression (n=4), vestibular dysfunction (n=3), and vision changes (n=3). Timing related to specific TB drug use was not reported. Sixteen (94%) patients received less than the approved initial dose of 1200 mg linezolid daily, and 15 (88%) patients underwent monitoring of linezolid exposure. All 16 patients with *M. tuberculosis* in initial sputa converted to negative culture results within 6 months of starting treatment. At 12 months after BPaL initiation, all patients had completed treatment, without TB recurrences or deaths reported.

**Conclusion:**

In the early period after FDA approval, most U.S. patients received BPaL off-label with an initial linezolid dose lower than the approved 1200mg yet still achieved good outcomes. Most reported patients underwent some monitoring of linezolid exposure. Monitoring of BPaL use is important and should continue.

**Disclosures:**

**All Authors**: No reported disclosures

